# Thermoforming for Small Feature Replication in Melt Electrowritten Membranes to Model Kidney Proximal Tubule

**DOI:** 10.1002/adhm.202401800

**Published:** 2024-11-07

**Authors:** Marta G Valverde, Claudia Stampa Zamorano, Dora Kožinec, Laura Benito Zarza, Anne Metje van Genderen, Robine Janssen, Miguel Castilho, Andrei Hrynevich, Tina Vermonden, Jos Malda, Mylene de Ruijter, Rosalinde Masereeuw, Silvia M Mihăilă

**Affiliations:** ^1^ Department of Pharmaceutical Sciences Div. Pharmacology Utrecht University Utrecht 13102 The Netherlands; ^2^ Department of Biomedical Engineering Technical University of Eindhoven Eindhoven 5612 The Netherlands; ^3^ Institute for Complex Molecular Systems Eindhoven University of Technology 5600 MB Eindhoven Eindhoven 513 The Netherlands; ^4^ Department of Orthopaedics University Medical Center Utrecht Utrecht 100 The Netherlands; ^5^ Department of Clinical Sciences, Faculty of Veterinary Medicine Utrecht University Utrecht 3584 The Netherlands; ^6^ Division of Pharmaceutics, Utrecht Institute for Pharmaceutical Sciences Utrecht University Universiteitsweg 99 Utrecht CG 3584 The Netherlands

**Keywords:** curvature, melt electrowriting (MEW), proximal tubule (PT), thermoforming, kidney tissue engineering, topography

## Abstract

A novel approach merging melt electrowriting (MEW) with matched die thermoforming to achieve scaffolds with micron‐sized curvatures (200 – 800 µm versus 1000 µm of mandrel printing) for in vitro modeling of the kidney proximal tubule (PT) is proposed. Recent advances in this field emphasize the relevance of accurately replicating the intricate tissue microenvironment, particularly the curvature of the nephrons’ tubular segments. While MEW offers promising capabilities for fabricating highly and porous precise 3D structures mimicking the PT, challenges persist in approximating the diameter of tubular scaffolds to match the actual PT. The thermoformed MEW membranes retain the initial MEW printing design parameters (rhombus geometry, porosity > 45%) while accurately following the imprinted curvature (ratios between 0.67‐0.95). PT epithelial cells cultured on these membranes demonstrate the ability to fill in the large pores of the membrane by secreting their own collagen IV‐rich extracellular matrix and form an organized, functional, and tight monolayer expressing characteristic PT markers. Besides approximating PT architecture, this setup maximizes the usable surface area for cell culture and molecular readouts. By closely mimicking the structural intricacies of native tissue architecture, this approach enhances the biomimetic fidelity of engineered scaffolds, offering potential applications beyond kidney tissue engineering.

## Graphical abstract

The combination of melt electrowriting and thermoforming allows to create porous membranes with precisely controlled microfeatures, including curvature and pore geometry. Proximal tubule cells are cultured on membranes with varying curvatures to investigate the impact of topographical cues on cell behavior and key cellular functions. This innovative approach enhances the structural and functional fidelity of kidney *in* vitro models.

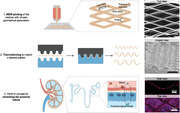

## 1 Introduction

As we gain a deeper comprehension of the intricate interactions between cells and the surrounding microenvironment, recapitulating this connection is required to study healthy and damaged tissues. The quest for accurately recreating complex architecture of biological tissues in vitro has led to the development of novel technologies/methodologies that seamlessly blend (sub)micro‐ and macro‐scale control. Within the available additive manufacturing techniques, melt electrowriting (MEW) allows fabrication of highly reproducible 3D structures with fibers that show diameters in the (sub‐)micrometer scale range.^[^ ^1^, ^2^ ^]^ Briefly, in MEW, a polymer is melted and electrostatically drawn onto a collecting plate, in a precise and tailored manner, enabling the fabrication of intricate fibrous scaffolds.^[^ ^3^ ^]^ In tissue engineering and regenerative medicine (TERM), the customization of scaffold architecture at the microscale is crucial,^[^ ^4^, ^5^, ^6^ ^]^ and the fine continuous fibers, high surface‐to‐volume ratio, and tunable pore dimensions and pore interconnectivity^[^ ^7^ ^]^ that can be achieved with MEW, makes it a valuable technology to mimic the topological characteristics of the extracellular matrix (ECM).^[^ ^2^ ^]^

In the field of kidney TERM specifically, MEW scaffolds are increasingly used due to their unique properties to guide cell behavior.^[^ ^8^, ^9^, ^10^, ^11^ ^]^ For example, MEW tubular scaffolds provide a self‐supported, functional replica of the proximal tubule (PT), in which the epithelial cells (PTECs) aligned differentially in response to the topographical guidance delivered by pore geometry.^[^ ^9^ ^]^ Notably, the PTECs and their secreted ECM (predominantly collagen IV) formed the barrier between the lumen and outside of the tube, thereby more closely mimicking the PT's micro‐ and macro‐environment. Other technologies to replicate the barrier have been studied as well, including the use of electrospun membranes with PTECs.^[^ ^8^, ^12^, ^13^, ^14^, ^15^ ^]^ However, the random fiber alignment and densely packed membranes in these models hamper studying transport of solutes and electrolytes, key functions of the PT, as these electrospun membranes represent a barrier on their own.

The PT presents a distinct set of challenges due to its intricate tubular structure, microscale features, and the necessity for a biomimetic environment. Amongst these features, curvature is emerging as a key topographical attribute to stir cell behavior and function.^[^ ^9^, ^16^, ^17^, ^18^ ^]^First attempts of linking PTEC function and curvature on silicon substrates highlight substantial differences between flat, concave, and convex curvatures, and curvature radii.^[^ ^16^, ^18^, ^19^ ^]^ For PTECs, both curvatures increase the expression of polarization markers, tight junction proteins, and preferential cytoskeletal arrangements appear. Despite there is, as of yet, no consensus on the preferred curvature, it is clear that it plays a key role in cell function. Current TERM approaches often fall short in simultaneously achieving the required fibrous architecture, microgeometry, and curvature, essential for mimicking the intricate physiological conditions of the PT.

Here, we introduce a pioneering approach that leverages the synergistic potential of MEW and matched die thermoforming for the advancement of PT‐TERM. Thermoforming implies warming up a polymer to achieve a plastic, yet solid stage, which allows the imprinting of a pattern.^[^ ^20^ ^]^ More specifically, micro matched die molding uses a combination of matched concave and convex details to imprint the features with high resolution and in one step. Variations of thermoforming have been combined with electrospinning techniques to create microwells from electrospun flat membranes. The resulting biocompatible scaffold enables free flow of nutrients and solutes while providing a microenvironment that sustained the growth of the tissue engineered islets of Langerhans,^[^ ^21^ ^]^ hepatocarcinoma spheroids^[^ ^22^ ^]^ and kidney organoids.^[^ ^23^ ^]^

With the aim to replicate the PT, the thermoforming molds were patterned in a half‐pipe shape geometry of different diameters to generate complex 3D‐patterned membranes with precise control over their structural properties while also supporting cellular function. The thermoformed MEW membranes maintain high permeability, enabling the diffusion of gas and nutrients, and the printed geometry stirs cell behavior by means of topographical guidance. Besides micro‐ and macro‐ architecture, this setup maximizes the usable surface area for cell culture and molecular readouts. Focused on the unique requirements of the PT, our method converges the benefits of MEW to produce intricate fibrous membranes with the ability of matched die thermoforming to impart microgeometry and curvature to thin films. The level of customization achieved by the combination of the two technologies holds great potential for mimicking the native environment of kidney tissues.

## 2 Results and Discussion

### 2.1 Optimized Micro Die Matched Thermoforming Allows Small Feature Imprinting in MEW Membranes

Until now, engineered PT replicates using MEW as a scaffold have been developed in the form of a tube with a diameter of 1 mm, over ten orders of magnitude above the native tubular diameter.^[^ ^9^, ^24^ ^]^ Tubular scaffolds with larger diameters have also been proposed for vascular replicates.^[^ ^25^, ^26^, ^27^ ^]^ While we have previously printed 500 µm diameter tubes, changes in working distance between the mandrel and the nozzle impaired fiber alignment and, thereby, yielded tubular scaffolds with non‐defined geometries. Aiming at approximating the original structure for unveiling direct connections between curvature and function, several grooved molds with diameters of 800, 600, 400, and 200 µm were designed for imprinting curvature in flat MEW membranes via matched die thermoforming.

**Figure 1:**
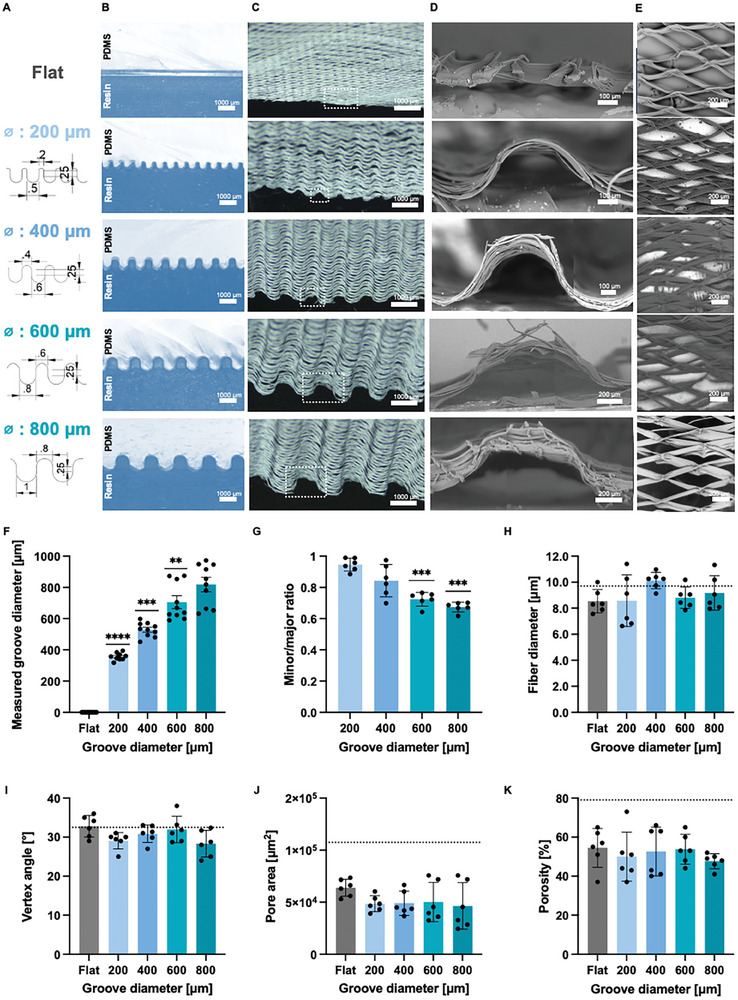
Optimized micro die matched thermoforming allows small feature imprinting in MEW membranes. A) Schematics on the CAD designs for the different groove sizes. Images of the paired PDMS and resin grooved molds (B) and the thermoformed MEW membranes (C). D) High magnification images (acquired with SEM) of the thermoformed membranes side views (composite of adjacent pictures) and top views (E). Measurements of the groove diameters (F) and the circularity of the grooves as minor/major ratio (G). The asterisks indicate the p values of comparison between the data and the target diameters, and the data and perfectly circular grooves (minor/major ratio = 1), respectively. Other membrane design parameters characterized include fiber diameter (H), vertex angle (I), pore area (J), and porosity (K) for the different patterns. Flat are membranes thermoformed with flat molds. Dotted lines indicate the value before thermoforming. Mean ± SD, *N* = 6, *p < 0.1, **p < 0.01, *** *p* < 0.001, **** *p* < 0.0001.

First, a variety of mold designs with different groove patterns were printed and tested to ensure reproducibility and accurate translation of the groove features into the MEW membranes (Figure S1, Supporting Information). Results showed that (1) it was not possible to eliminate the spacing between grooves, (2) the grooves needed a certain height (here optimized to 250 µm), (3) sharp edges were not translated accurately from the computer‐aided design (CAD) to the printed digital light processing (DLP) model and (4) concave spacings required a greater diameter in the CAD for the reproducibility of the patterns (convex diameter plus 200 µm or 300 µm). Accounting for these considerations, the CAD models for the negative resin molds were designed without sharp edges, thus the final molds have both concave and convex grooves in a wave pattern (**Figure** **1**A). The resulting resin molds accurately maintained the desired groove features, which were successfully translated to the polydimethylsiloxane (PDMS) molds via soft lithography (Figure 1B). The combination of a soft (PDMS) and a stiff phase (resin) improves the feature transferring in electrospun membranes.^[^ ^22^ ^]^

Flat poly‐ε‐caprolactone (PCL) MEW membranes, with an internal rhombus geometry, were successfully printed and characterized with SEM before the processing (Figure S2, Supporting Information). The vertex angle was 32.5 ± 1.7, fiber diameter 9.7 ± 0.7 µm, pore area 108,000 ± 12,000 µm^2^, and porosity 79 ± 0.06% (mean ± SD; Figure S3, Supporting Information). Microthermoforming requires heating of the polymer films to a softened, yet still solid state before the imprinting. Besides enabling the plastic deformation, it renders high material coherence implying that pre‐modifications of the material (e.g., MEW fibers) are maintained.^[^ ^20^ ^]^ PCL MEW rhombus membranes allow for maintaining microfeatures such as winding angles, which we aim to preserve for steering cell behavior. PCL is a semicrystalline polymer with a low melting point of ≈60 °C and a glass transition temperature of ‐60 °C.^[^ ^28^ ^]^ Pilot studies carried out during the development of the protocols revealed that temperatures above 55 °C cause membrane melting (data not shown), therefore, setting the processing temperature. Optimized resin and matching PDMS molds, and temperature conditions allowed for the indentation of the grooves from 200 to 800 µm in diameter in the MEW membranes (Figure 1C–E). The thermoformed membranes were produced with diameters higher than expected, between 357and 819 µm, respectively (Figure 1F). To characterize how accurately the membranes trace the profile of the curve (amplitude and height), the parameter *minor to major ratio* was defined. Curvature was well reproduced in all cases, with *minor to major ratios* between 0.67 and 0.95 (Figure 1G). As expected, the thermoforming process slightly elongated and flattened the MEW fibers, which did not cause a significant change in the fiber diameter (Figure 1H). Regarding the geometry of the pores, the vertex angle was maintained in different regions of the curve (Figure 1I), whereas the pore size and porosity diminished with the thermoforming (Figure 1J,K). The thermoforming process causes compression of the walls that form the 3D scaffold (Figure S4, Supporting Information), reducing the pore size and porosity. As previously reported, the shift that vertical structures undergo can be up to 50 µm.^[^ ^20^ ^]^ Smaller filament diameters or less number of printed layers partially solved the closing of the pores but made the handling and assembly in Transwells for cell culture not possible (data not shown).

Besides approximating the curvature of the membranes to that of the native PT, patterning increases the effective surface area for cell culture, which is key for sample collection and analysis. Larger culture areas are necessary for obtaining sufficient samples of cell culture medium or cellular products for analysis. This is particularly important for studying the release of biomarkers, cytokines, or other molecules by cells in response to various stimuli in the field of kidney‐on‐chip, where the areas of culture are generally very limited. Assuming that the whole surface of the membrane is comprised by equal grooves, the mathematical description of the surface area gain (Equation 4) describes this is only dependent on the ratio between the minor and major axis of the grooves for the patterned membranes. The available area of flat membranes remains unaltered. Applying the formula to the experimental measurements of the thermoformed MEW membranes, it is possible to quantify the actual surface area gain which is gained for the same space: 53.5% for the 200 µm membrane, 39.5% for the 400 µm membrane, 36.1% for the 600 µm membrane and 33.5% for the 800 µm membrane (Figure S5, Supporting Information).

Grooved membranes for miniaturization, are not the only possible design. As a proof of concept, we designed molds with half spheres, a horse‐shoe shape, and channels with changing diameters. The results (in Figure S6, Supporting Information) support the versatility of our technique. The half spheres are comparable with microwells and can be useful for the culture of lobular epithelium such as the alveoli or breast with the advantage of tunable geometries for meeting the tissue's demands.^[^ ^21^, ^23^ ^]^

### 2.2 PTECs Deposit Their Own Extracellular Matrix within the Pores of the Thermoformed MEW Membranes

**Figure 2:**
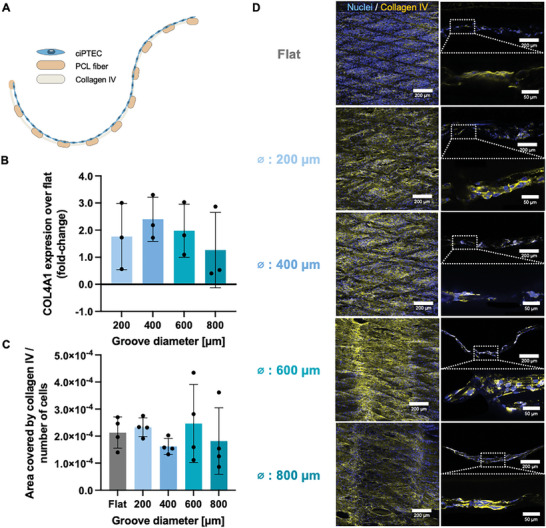
Collagen IV deposition on the pores of MEW membranes allows the establishment of a BM which cells use for bridging the pores. A) Schematic on the disposition of cells, collagen IV BM and PCL MEW fibers. B) Relative mRNA expression of collagen type IV of thermoformed MEW grooved membranes compared to thermoformed MEW flat membranes. C) Quantification of fluorescence intensity of the immunofluorescent images calculated over the number of cells and corrected for surface area gain. D) Immunofluorescent images of collagen type IV (yellow) and DAPI (blue) in MEW flat and grooved membranes, from the top and the side (images acquired with 25x objective). The dashed squares represent the zoomed in areas (images acquired with 63x objective). Mean ± SD, *N* = 3 for COL4A1 and *N* = 4 for image quantification.

The prerequisite for creating functional bioengineered kidney membranes is the adherence and growth of cells to form a monolayer that acts as a barrier. Previous results demonstrated that conditionally immortalized PTECs (ciPTECs) can bridge the 200 µm pores in functionalized PCL microfibers in tubular scaffolds.^[^ ^9^, ^12^ ^]^ Like in tubular scaffolds, the cells seeded on thermoformed membranes migrated from the vertex of the pores toward the center whilst secreting a basal membrane (BM) (**Figure** **2**A; Figure S7, Supporting Information). On mRNA level, we observed a slight increase in collagen IV expression in the grooved membranes compared to the flat membranes (fold‐change over flat between 1.2‐2.4, p = 0.36) (Figure 2B). The production of a collagen IV network occurred for all membranes, independently of the groove size (Figure 2C), and covers the otherwise empty space within the pore (Figure 2D). We hypothesize that the relative increase in the expression of collagen IV can be related to the curvature.

Other studies using PT‐on‐chip with flat membranes demonstrated that PTECs produce a collagen IV‐rich BM.^[^ ^29^, ^30^ ^]^ However, a link between COL4A1 mRNA expression and collagen protein deposition with curvature has not been reported yet. For instance, the thorough study by van Gaal et al. for elucidating the response of PTECs to curvature did not report collagen IV.^[^ ^16^ ^]^ Neither did Yu et al., who functionalized their curved surface with collagen IV to induce cell attachment.^[^ ^18^ ^]^ Additional assays like exposure to TBF‐ß or a knock‐down of precursors involved in the synthesis of fibrillar collagen could shed further light on the mechanisms triggering collagen production.^[^ ^31^, ^32^ ^]^ Recently, flow has been reported to induce the reorganization of the collagen network deposited by podocytes, another kidney epithelial cell type.^[^ ^33^ ^]^

### 2.3 PTECs Form Organized Monolayers Covering the Pores and following the Curvature of the Thermoformed MEW Membranes

The deposition of collagen IV is only possible as the PTECs span across the pores of the scaffold. Likewise, ciPTECs can bridge across non‐adhesive areas of the scaffold because of their epithelial nature in cell‐to‐cell contact organization, but also by the characteristics of the scaffold.^[^ ^34^ ^]^ We hypothesize that it is a set of topographical cues that enables the initial bridging that culminates with the establishment of a cell monolayer. As such, the small diameter of the fibers, the smooth surface of the MEW fibers, and the pores that are not too large are key determinants.

**Figure 3:**
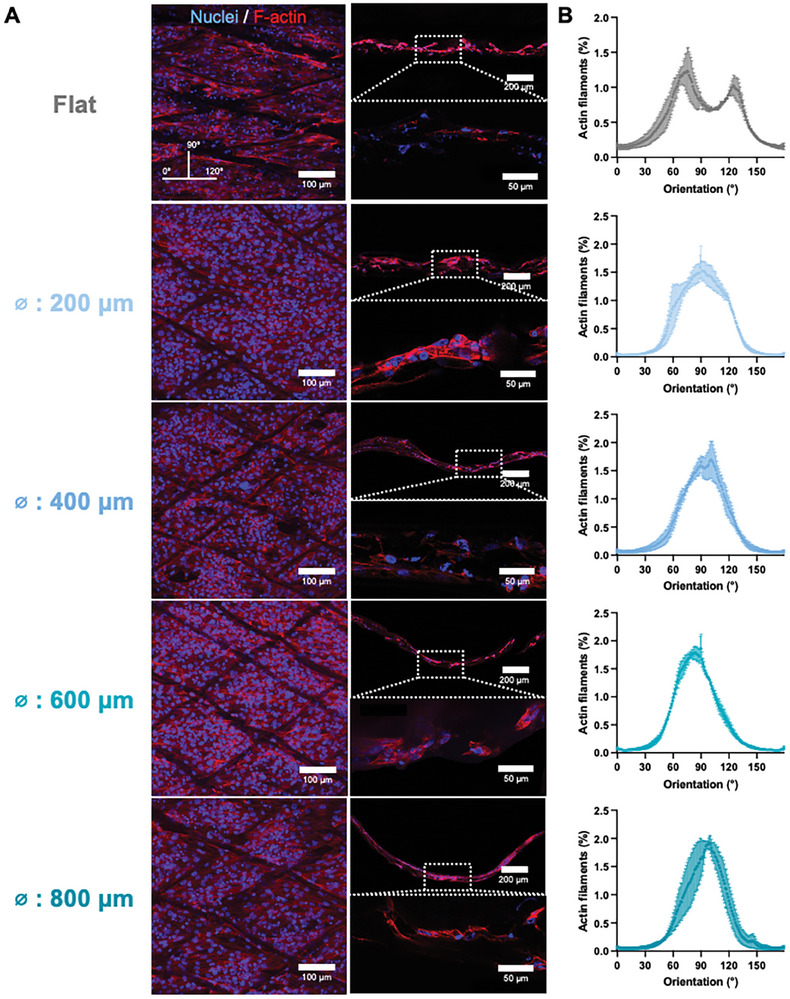
ciPTECs form an organized monolayer across the pore. A) Immunofluorescent images of actin filaments (red) and nuclei (blue) in MEW flat and grooved membranes from the top and the side (images acquired with 25x objective). Dashed squares represent the zoomed in areas (images acquired with 63x objective). B) Quantification of cell directionality in MEW flat and grooved membranes.

Staining of the cell nuclei and cytoplasm (F‐actin) confirmed that cells grew into a monolayer across the pores (**Figure** **3**A). Next, cell directionality and arrangement were studied for the different groove sizes. On the grooved membranes, the cytoskeleton filaments were preferentially aligned with the major diagonal of the rhombus pore and, thus, perpendicularly to the groove (90°, Figure 3B). However, the organization of the flat membranes is not as clear, with two peaks, ≈70° and 130°. It is possible to speculate that the curvature is supporting a circumferential alignment, similarly to other PT models.^[^ ^35^ ^]^

Thus, we enable a semi‐scaffold‐free approach, where the large pore configuration permits cells to establish suspended monolayers sustained by the in situ deposited ECM. This methodology not only facilitates the recapitulation of the biological microenvironment, but also minimizes interference from the MEW material, in functional assays (eg. transepitelial transport of molecules). In the end, the MEW membrane primarily serves as a structural frame and the cells are the sole barrier separating apical and basal sides.

### 2.4 The PT Engineered Membranes are Leak‐Tight and Exhibit PT Polarization Markers

**Figure 4:**
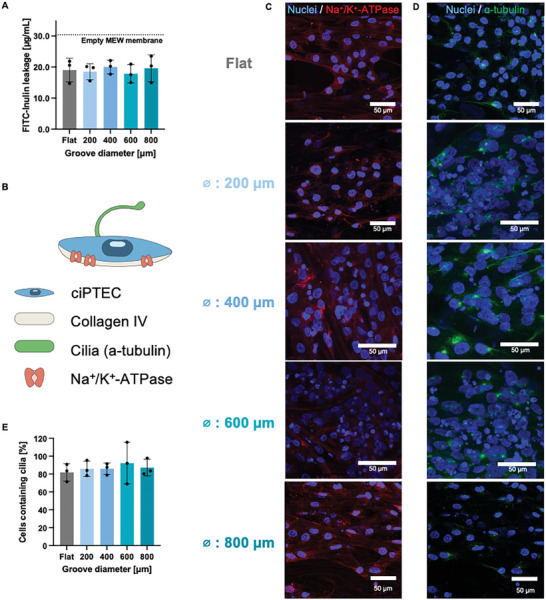
PT engineered membranes are leak‐tight and exhibit PT polarization makers. A) Quantification of inulin‐FITC leakage from the apical to the basal compartment of thermoformed MEW grooved membranes normalized to the leakage of thermoformed MEW flat membranes – both containing cells. B) Schematic representation of the polarization markers in the ciPTEC line. C) Immunofluorescent images of Na^+^/K^+^ ATPase (red) and cell nuclei (blue) proving basal polarization. D) Likewise, positive α‐tubulin (green) and nuclei (blue) showcase apical polarization for all MEW membranes. E) Quantification of the number of cells containing cilia. Mean ± SD, *N* = 3.

The barrier formed by the cells and their self‐assembled BM must be leak‐tight as in the native PT. To confirm the barrier functionality of the monolayer formed in the thermoformed membranes, apical‐to‐basal FITC‐inulin leakage was measured. Similarly to the findings reported previously for tubular scaffolds, the cell monolayer and the secreted BM form a tight barrier that is impermeable to inulin‐FITC diffusion in the upper compartment of the Transwell system.^[^ ^9^ ^]^ All curved membranes showed less leakage compared to the thermoformed empty membranes (**Figure** **4**A), and similar values as the flat membranes. No differences in leakage were found amongst groove sizes. Remarkably, the membranes’ filtration properties are cell‐sustained, differing from other proposed membranes (like PDMS) which are known for retaining small molecules.^[^ ^36^ ^]^ In the context of PT‐on‐chip, the MEW thermoformed membranes are also compatible with molecules that otherwise cannot be assesed because they are larger than the cuttoff size of the membrane (PDMS, microPES).^[^ ^36^, ^37^ ^]^

When establishing a confluent monolayer, ciPTECs express distinct polarization markers.^[^ ^35^, ^38^, ^39^ ^]^ Until date, the presence of these markers in MEW membranes had not been reported. Na^+^/K^+^ ATPase and α‐tubulin appear basally and apically expressed, respectively (Figure 3B). For all groove sizes, cells exhibited polarization markers confirming the establishment of an epithelial monolayer and a response to apical‐basal guidance (Figure 3C,D). Furthermore, the quantification of the number of cilia proved that 81.9 ± 5.5% of the cells in flat membranes express cilia, and 87.8 ± 3.5% for the cells grown on grooved membranes.

It is important to note that imaging the polarization markers (Na^+^/K^+^‐ATPase and α‐tubulin) in the MEW scaffolds is technically challenging. Due to the need for higher magnifications, the focal depth is reduced, significantly limiting the visible area and the depth of the images. This limitation persisted even when the membranes were assembled perpendicular to the objective (Figure S8, Supporting Information). Alternative methods for processing the membranes should be investigated to assess the precise location of these markers. Therefore, we relied on the validation of polarization through functional assays, which provide a more comprehensive assessment of the cells’ polarized behavior, compensating for the limitations in direct imaging of the markers.

### 2.5 The PT Membranes Show Transporter Function and Expression

The kidney is responsible for blood filtration to excrete toxins and toxicants, maintaining acid–base and body volume homeostasis.^[^ ^40^, ^41^ ^]^ Within the nephrons of the kidney, tubular reabsorption begins as the filtrate enters the PT and involves the re‐uptake of nutrients and ions coupled with passive water reabsorption.^[^ ^42^ ^]^ Moreover, the PT has an essential function in active waste secretion, from blood to tubular lumen. We investigated the presence and functionality of two efflux pumps: breast cancer resistance protein (BCRP) and multidrug resistant protein 4 (MRP4), both involved in the transport of organic solutes, including drugs, from the PTEC to the tubular lumen^[^ ^43^, ^44^ ^]^ The combined activity of BCRP and MRP4 is integral to the function of the renal excretion machinery for maintaining homeostasis and can be tested by the secretion of calcein, a negatively charged organic compound derived from calcein‐AM that enters the cells by diffusion and is hydrolyzed into its fluorescent form.^[^ ^35^, ^45^ ^]^

**Figure 5:**
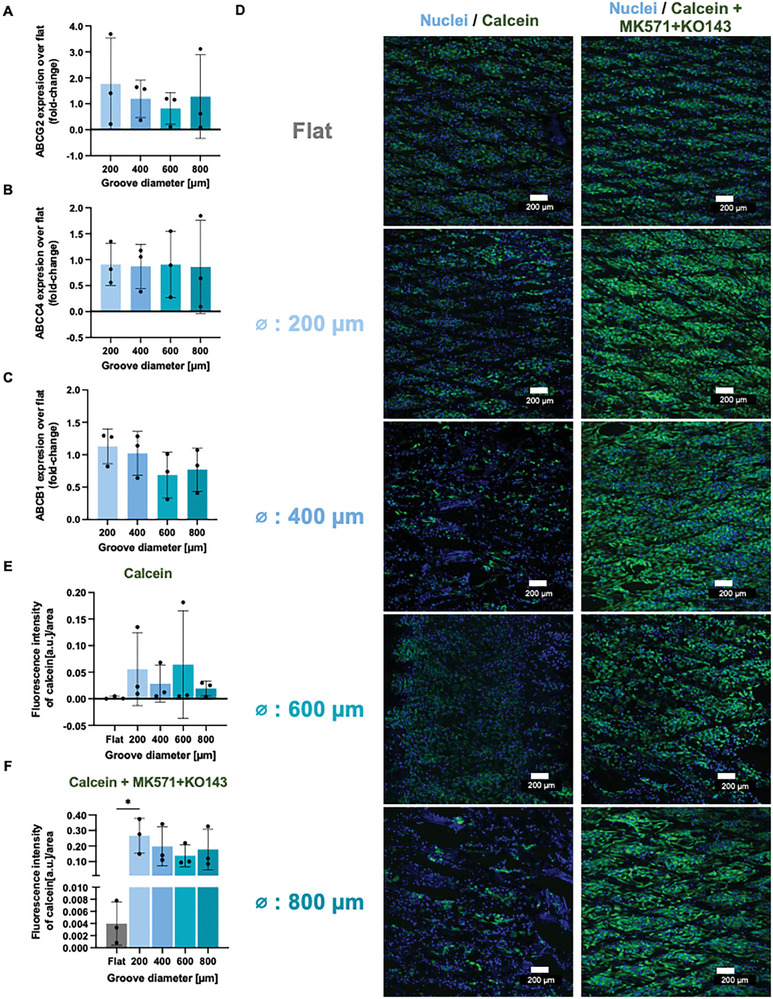
PT engineered membranes demonstrate renal transporter expression and function. (Relative mRNA expression of BCRP (A), MRP4 (B), and P‐gp (C) of ciPTECs on thermoformed MEW grooved membranes compared to thermoformed MEW flat membranes. D) Immunofluorescent images of PTECs retaining calcein in absence (left) or presence (right) of inhibitors. Quantification of the intensity of the immunofluorescent images calculated over the imaged area and corrected over surface area gain without (E) and with (F) inhibitors. RT‐qPCR *N* = 3, with each n consisting of at least 5 membranes pooled for the same condition. Mean ± SD, *N* = 3, *p< 0.1, **p < 0.01, *** *p*< 0.001, **** *p* < 0.0001.

The presence of the transporters was quantified by the mRNA expression of BCRP (*ABCG2*) and MRP4 (*ABCC4*) (**Figure** **5**A,B, respectively). In addition, P‐glycoprotein (P‐gp, *ABCB1*), another apically expressed ATP‐dependent transporter, was evaluated (Figure 5C). Despite an increase in BCRP expression compared to flat membranes, no differences in the expression of any of the three transporters were observed for the various curved membranes tested. However, in terms of their functionality, an increase in intracellular calcein accumulation was observed upon addition of transport inhibitors MK571 and KO143 that block BCRP and MRP4, respectively (Figure 5D). After correction for the surface area gain, quantification of the images shows that cells on curved membranes retained more calcein (Figure 5E) and are more sensitive to the presence of inhibitors (Figure 5F).

The gene expression data has high variability, in part, because of the isolation process. Despite the increased surface area for culture, several membranes needed to be pooled for obtaining sufficient RNA. Other techniques with higher sensitivity could offer new insights in the genetic profile of cells cultured on different grooves. Despite introducing curvature in the system, more stimuli are required to enhance the expression of markers. Fluidic shear stress (FSS) is also known to boost transporter function in epithelial cells.^[^ ^46^ ^]^ Consequently, the next step would be to make use of the designed chip to incorporate FSS and analyze whether shear stress can indeed increase transporter expression.

## 3 Conclusions

Until date, artificial membranes to replicate barrier tissues ‐like the kidney tubule epithelium‐ have been based on polymeric materials (PDMS and polycarbonate) with small pores.^[^ ^36^, ^47^ ^]^ Despite tunable pore sizes and porosities, and the imprinting of nanofeatures, the main barrier between the two compartments is the membrane itself, limiting the cellular contribution.^[^ ^48^ ^]^ The inert materials used do not only hamper the functional remodeling of the ECM, but are also known to absorb small molecules, reducing their applicability for drug testing.^[^ ^49^ ^]^ Moreover, these membranes are usually flat, which does not recapitulate the native geometry of the PT epithelium. Recent literature highlights the importance of topographical features for guiding PT cell behavior. On the micro scale, (bio)printed tubular hydrogel scaffolds are able to recapitulate a lumen with diameters of ≈250 µm.^[^ ^35^, ^50^, ^51^ ^]^ Yet, transport studies across the cell monolayer are challenging as the gels do not allow for free diffusion of molecules toward the basal compartment. Alternatively, MEW tubes can be applied for PT replicates as previously proposed by us. These leverage high porosity and tunable pore sizes and geometries to guide cell behavior.^[^ ^9^ ^]^ Yet, their inner diameter (1 mm) is far from the native dimensions. Printing smaller tubes on a mandrel‐based system is challenging. Approaching smaller diameters and overcoming the mandrel limitations, we propose here the convergence of MEW and thermoforming for imprinting micrometrical features on MEW membranes.

During a previous pilot study, we investigated the possibility of printing directly on top of several curved collectors of various materials (resin, PDMS, aluminum foil, stainless steel, and needles). The results, summarized in Figure S9 (Supporting Information), reveal compatibility issues between the MEW and micro‐curved collectors, as the fibers appear randomly aligned or not following the curvature. Either the repelling electrostatic properties of the material and/or the machine resolution impaired the reproduction of micro features while maintaining geometrically defined structures. Overcoming the resolution limitations, we propose the convergence of micro die matched thermoforming, a post processing method, with accurately printed MEW membranes for enabling the creation of a new dimension of topographical features.

First, we designed and optimized a series of matched stamps featuring cylindrical grooves ranging from 200 µm to 800 µm in diameter. Harnessing the resolution and versatility of DLP 3D printing and soft lithography, molds accurately following the computer designs were created. The fabrication of the molds and the transfer to PDMS can be improved further with other fabrication techniques with superior resolution, such as photolithography.^[^ ^16^ ^]^ This might allow imprinting smaller feature sizes and to reduce the sacrificial curvature needed for transferring the pattern. Reducing the sacrificial curvature would also imply having only one curvature: either convex or concave (as attempted initially and described in the optimization). The application of thermoforming rendered MEW membranes which accurately and non‐reversibly follow the curvature of the molds while maintaining key features like fiber diameter and vertex angle. These topographical features are hypothesized to have a strong impact on cell behavior.^[^ ^9^ ^]^ However, as part of the imprinting process, the membrane's vertical walls appeared displaced as they accommodate the new curvature, thus, reducing the pore size and porosity from a 60% to ≈40%. Besides replicating the curved topography of the PT, an additional advantage of the grooved membranes is the increased surface area, which can ultimately be beneficial for readouts that require high(er) number of cells.

After printing, the patterned MEW membranes were assembled in Transwells and prepared for cell culture. ciPTECs were able to form confluent, leak tight monolayers, showing preferred spatial alignment, secrete their own collagen IV‐containing extracellular matrix, express polarization markers, and demonstrate active transport sensitive to specific inhibitors. We have previously shown the geometry instruction effect on tubular scaffolds with different pore shapes (rhombus versus square versus random).^[^ ^9^ ^]^ With our new approach, it is possible to also confirm that cell alignment did not vary with the groove diameter but is mostly dependent on the topographical guidance given by the direction of the PCL fibers.

Overall, this study proposes an innovative application of thermoforming for an advanced in vitro model of the PT. While increasing the usable surface area of the substrate, the MEW patterned membrane simulates the physiological environment of the cells by mimicking the fibrous and porous structure of the ECM and by introducing micro scale curvature. By leveraging the advantages of MEW and thermoforming, this method can be applied to a plethora of tissues whose architecture replica in vitro has been challenging up to date, indicating exciting opportunities for advanced tissue strategies. The bioartificial kidney is one of the candidates that could benefit from the miniaturization offered by our membranes.^[^ ^11^, ^52^ ^]^ Reducing size while maintaining usable area for cell culture and filtration offers a promising alternative to the classical dialysis membranes. Beyond the kidney, any tissue could leverage having topographical guidance at two different levels (macro‐ and micro‐), including gut villi,^[^ ^53^ ^]^ branching vasculature,^[^ ^54^ ^]^ or alveoli.^[^ ^55^ ^]^ In our study, the membranes were attached to inserts and cultured in static conditions. However, recent advances on on‐chip technologies highlight the importance of culturing cells, and particularly kidney cells, under flow conditions for further recapitulating the native fluidic forces.^[^ ^35^, ^40^, ^46^, ^56^, ^57^ ^]^ Future directions of the thermoformed membranes include their incorporation on perfusable devices, where fluids flow parallel to the grooves, and their application to other tissues. The fields of disease modeling, drug testing, and personalized medicine could harness the more accurate microenvironment and enhanced versatility of the thermoformed MEW membranes compared to 2D cultures^[^ ^10^, ^58^ ^]^ to elaborate on the transport activity and other physiological responses. In conclusion, the combination of MEW and thermoforming provides a novel approach for tissue engineering. By addressing architectural challenges and enhancing microenvironment fidelity, these membranes pave the way for more accurate physiological responses and transformative medical applications.

## 4 Experimental Section

### MEW of Fibrous Membranes

MEW microfiber membranes were fabricated using a custom‐designed printer equipped with a flat collector plate, movable along the x and y axes and controlled via Motion Perfect v.5.0.2 (Trio Motion Technology, Tewkesbury, UK). Medical grade poly‐ε‐caprolactone (Purasorb PC 12, Corbion, Amsterdam, The Netherlands) granules were loaded into a glass syringe (Fortuna Optima, Poulten & Graf GmbH, Wertheim, Germany), melted at 100 °C, and extruded through a 25G nozzle. With an air pressure of 0.2 MPa, the Taylor cone was established by applying 5 kV voltage at a 3 mm collector distance. Fibers were collected at a speed of 7 mm^−1^s. Prior to printing the actual design, ten stabilization lines were deposited to ensure stable reproducible fiber diameters. The printing pattern of the fiber membrane was communicated via G‐code, and the internal membrane design featured rhombus shapes with a pore size of 200 µm and a 30° vertex angle. Constructs were printed with 15 layers and the total dimension of the membrane was 80 × 80 mm.

### Patterned Molds

**Figure 6:**
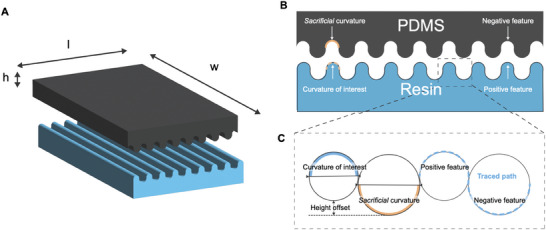
Schematic of the patterned molds. A) Example of the 3D rendering of the CAD design for the 800 µm mold. B) Front view of the molds and the design parameters. C) The curvature of interest appears as the positive feature in the resin, while the sacrificial curvature appears as the negative.

Complementary positive (resin) and negative (silicon) molds were created to imprint the curvature on flat MEW membrane (Figure 5). The mold designs were created using Autodesk Fusion 360 (Autodesk Inc, San Francisco, CA, USA) software. To create the MEW grooved membranes, four molds, each measuring ≈2 cm × 2 cm × 0.5 cm (l × w × h), were designed with alternating concave and convex grooves patterns as described in **Table** **1**. The final dimension of the mold varied depending on the number of grooves, but this did not affect the outcome (**Figure** **6**).

**Table 1:** Patterned mold design parameters.

Curvature of interest [µm]	Sacrificial curvature [µm]	Height offset [µm]	Number of grooves
200	500	250	29
400	600	250	20
600	800	250	14
800	1000	250	11

The 3D models were exported into. stl files and imported into a DLP 3D printer (EnvisionTEC, Gladbeck, Germany). The molds were printed with an 85 mm focal lens using a PIC100 resin (EnvisionTEC). After printing, the molds were sonicated twice in ethanol 100% for 10 min and post‐cured under 3 000 light flashes (BEGO Otoflash G 171, Lincoln, RI, USA) to remove any uncured material. Then, by casting PDMS (SYLGARD™ 184 Silicone Elastomer Kit, Dow, Midland, MI, USA) onto these positive resin molds, patterned negative molds were created. The uncured PDMS mixture was prepared according to the manufacturer's instructions, degassed for at least 15 min in a vacuum chamber after pouring, and cured for 4 h at 80 °C.

### Matched Die Thermoforming of MEW Membranes

Flat 2 cm x 2 cm MEW rhombus membranes were heated at 55 °C in a convection oven for 5 min and placed in between a pair of matching molds of PIC100 resin and PDMS. Afterward, 45 Pa of pressure were applied on top of the PIC100 resin mold. After 1 min, the load was lifted, and the thermoformed MEW patterned membrane was removed from the molds. The thermoforming process was done for flat and grooved molds to obtain thermoformed flat and grooved membranes. Flat thermoformed MEW membranes were used as control.

### Membrane Characterization

Scanning electron microscopy (SEM, Phenom Pro, Phenom World‐ThermoFischer Scientific, Waltham, MA, USA) was employed to image the MEW membranes before and after thermoforming. The membranes were sputter coated in platinum (6 nm thickness) prior to imaging using a rotary‐pumped sputter (Quorum Technologies, Lewes, UK). The obtained images were analyzed using ImageJ software (National Institute of Health, US) to measure the different macro‐ and micro‐architectural features (Figure S2, Supporting Information). For display purposes, the cross‐section images of the thermoformed membranes are composed of adjacent pictures.

### Surface Area Gain Calculation

The percentage of surface area gain of the grooved membranes was defined as:

(1) $$\%\;Surface\;Area\;gain=\frac{Surface\;Area_{groove}-Surface\;Area_{flat}}{Surface\;Area_{flat}}\;*100$$

where *Surface* *Area*_*flat*_ was the surface of a flat membrane, and was calculated as,

(2) $$Surface\;Area_{flat}=l*w$$

with *l* being the length of the membrane and *w* being the width of the membrane; and where *Surface* *Area*_*groove*_ was the surface area of a grooved membrane, and was calculated as

(3) $$Surface\;Area_{groove}\left(\sqrt{\frac{\left(1+x^{2}\right)}{2}}\right)*\left(\frac{l*w*\pi }{2}\right)$$

with *l* being the length of the membrane, *w* being the width of the membrane and *x* being the ratio between the minor and major axis of the groove. By plugging equation (Equation 2) and (Equation 3) into equation (Equation 1), the resulting equation was Equation 4. Further explanations are included in Figure S5 (Supporting Information).

(4) $$\%\;Surface\;Area\;gain=\left[\left(\frac{\pi }{2}\right)*\left(\sqrt{\frac{\left(1+x^{2}\right)}{2}}\right)-1\right]\;*100$$

### Cell Culture

Human conditionally immortalized proximal tubule epithelial cells (ciPTECs 21.2) originally isolated from healthy human kidney were developed by Jansen et al.^[^ ^59^ ^]^ and currently provided by Cell4Pharma (Oss, The Netherlands; www.cell4pharma.com) with the ethical approval and quality assurance in place. Cells were cultured in PTEC culture medium and passaged as described previously,^[^ ^59^ ^]^ at 33 °C and 5% (v/v) CO_2_ up to 90% confluency for maintaining a cell proliferation state. For cycle arrest and maturation, ciPTECs were transferred to 37 °C for 7 days prior to experimental readout.

### Transwell Assembly and Membrane Biofunctionalization

Thermoformed MEW membranes were assembled in Transwell systems (Corning COSTAR, Corning, NY, USA). The original membrane of the Transwells was replaced with thermoformed MEW membranes by attaching a custom‐made polyether ether ketone (PEEK) ring to the bottom of the Transwells. The system was sterilized in 70% ethanol for 1 h, washed with HBSS (Gibco Hans Balanced Salt Solution, ThermoFisher Scientific, Waltham, MA, USA) 3 times for 5 min, and exposed to UV light (365 nm) for 20 min. A solution of L‐3,4‐ dihydroxyphenylalanine (L‐DOPA, 2 mg mL⁻^1^, Sigma Aldrich, St. Louis, MO, USA) in tris(hydroxyethyl)aminomethane (Tris, 10 mM) buffer pH 8.5 was prepared, dissolved at 37 °C for 1 h and filter‐sterilized for coating the membranes through submersion for at least 4 h at 37 °C. After removing the coating solution, the membranes were washed three times in HBSS 5 min and let to air‐dry inside the flow hood for at least 15 min before seeding the cells.

### Cell Seeding and Culture on Membranes

For cell seeding, the Transwells containing coated thermoformed MEW membranes were placed upside down in the lid of the plate. A cell suspension of 15 × 10^6^ cells/mL was prepared, and a 20 µL drop was laid on the bottom side of air‐dried membrane (facing upward at this stage). For maximizing cell attachment, the seeded plates were placed upside‐down in the incubator. After 1.5 h at 33 °C and 5% (v/v) CO_2_, the plates were flipped to the normal position, and after a total time of 3h, 1 mL of medium was carefully added to the bottom compartment of each Transwell. The following day, the membranes were transferred onto a new plate with new media to discard any unattached cells. The cells on the membranes were left to proliferate at 33 °C and 5% (v/v) CO_2_ for 7 days until visual inspection under the microscope confirmed the full pore coverage by cells. Last, the membranes were transferred for maturation to 37 °C and 5% (v/v) CO_2_ for 1 week and medium was refreshed bi‐weekly.

### Immunofluorescence Stainings

Cell coated membranes were washed with HBSS 3 times for 5 min, fixed for 20 min in 2% (v/v) paraformaldehyde (4% paraformaldehyde diluted 1:1 in cell culture medium) and permeabilized in triton X‐100 (0.3% (v/v)) in Phosphate Buffered Saline (PBS, ThermoFisher Scientific, Waltham, MA, USA) for at least 10 min. Afterward, the membranes were submerged in blocking buffer (BB, 2% (v/v) FCS, 0.5% (w/v) bovine serum albumin (BSA), and 0.1% (v/v) Tween20 in PBS) for at least 30 min. The primary antibodies (Table S1, Supporting Information) were diluted in BB and incubated at room temperature for at least 1 h. After washing the membranes 3 times for 5 min with Tween20 (0.1% (v/v)) in PBS, the secondary antibodies (Table S1, Supporting Information) diluted in BB were added, and incubated, protected from light, at room temperature for another hour. After washing, DAPI in BB was added to the samples, and incubated, protected from light, at room temperature for 8 min. Finally, the membranes were washed 3 times for 3 min with PBS and mounted in Wilco dishes (WillCo Wells B.V, Amsterdam, The Netherlands). Fluorescence images were obtained using confocal microscopy (Leica TCS SP8 X, Wetzlar, Germany) and analyzed using ImageJ. For the images from the top of the membrane, z‐projections were made. Actin filament directionality was quantified using the cell directionality functionality plugin of ImageJ, and fluorescence intensity was quantified by converting the images to 8‐bit and applying the same thresholds to all images.

### Leakage Assay

To assess the leak‐tightness of the mature monolayers on thermoformed MEW membranes, a leakage assay was performed using fluorescence isothiocyanate‐inulin (inulin‐FITC, Sigma‐Aldrich, St. Louis, MO, US). Mature membranes assembled on Transwells were rinsed with HBSS and 1 mL of media was added to the bottom compartment. 200 µL of inulin‐FITC (0.1 mg/mL) were placed on the top compartment of the membranes and after 20 min of incubation protected from light, 100 µL duplicate samples were taken from the bottom compartment of each well. The samples were read in the plate reader (GloMax Discover Microplate Reader, Promega, Madison, WI, USA) at 475 nm excitation and 500–550 nm emission, to quantify the fluorescence. The numerical results were corrected for surface area gain.

### Transepithelial Transport Assay

To assess the transporter functionality, mature cell‐loaded membranes assembled in Transwells were rinsed with HBSS and incubated with calcein‐AM (1 µM; Invitrogen, ThermoFisher Scientific, Carlsbad, CA, USA) loaded apically and basally at 37 °C for 15 min. For validating their functionality, half of the samples for all the groups were pre‐incubated for 15 min with the transporter inhibitors MK571 and KO173, for MRP4 and BCRP respectively (5 µM and 5µM; Sigma‐Aldrich, Saint Louis, MO, USA). After incubation, the membranes were rinsed with HBSS, and nuclei were stained with Hoechst 33342 (1:10000 in HBSS; Invitrogen, ThermoFisher Scientific, Carlsbad, CA, USA). After washing 3 times with HBSS, fluorescent images were obtained with confocal microscopy (Leica TCS SP8 X) and z‐projections were made. The fluorescence intensity was quantified using ImageJ (National Institutes of Health) after converting the images to 8‐bit and applying the same thresholds to all images. The numerical results were corrected for surface area gain.

### Gene Expression

Gene expression analysis was done via reverse transcription‐quantitative polymerase chain reaction (RT‐qPCR) on mature cells growing on thermoformed MEW membranes. After washing the membranes 3 times with HBSS, four membranes with the same groove size were pooled together. The cells were then re‐harvested by an incubation with Accutase (37 °C, 10 min), followed by a washing step of the pellet in HBSS. Then, RNA was isolated by following the protocol of the PureLink RNA Mini Kit (Invitrogen, ThermoFisher Scientific, Waltham, MA, USA) according to the manufacturer. After isolation, cDNA was prepared using the iScript cDNA Synthesis Kit (BioRad, Lunteren, The Netherlands) according to the manufacturer's instructions. RT‐qPCR analysis was completed in the CFX96 real‐time PCR detection system (BioRad, Lunteren, The Netherlands). TaqMan Universal MasterMix and TaqMan Gene Expression Assay (ThermoFisher Scientific, Waltham, MA, USA) were used for *COL4A1* (TaqMan Assay ID: Hs00266237_m1), *ABCB1* (Hs00184500_m1), *ABCC4* (Hs00988721_m1) and *ABCG2* (Hs01053790_m1). *RPS18* (Hs01375212_g1) was used as the reference gene. The gene expression fold change was calculated according to the 2 ^−ΔΔCt^ method.

### Statistical Analysis

Statistical analysis was performed in GraphPad PRISM (GraphPad Software Inc., La Jolla, CA, USA) using the one‐way ANOVA and Tukey's multiple comparison tests. Unless otherwise specified, a minimum of three individual experiments were performed. Differences were considered significant with a p‐value of p<0.1 and data were represented as a mean ± standard deviation (SD).

## Conflict of Interest

The authors declare no conflict of interest.

## Supporting information



Supporting Information

## Data Availability

The data that support the findings of this study are available from the corresponding author upon reasonable request.
