# Supplementary material for: Thermoforming for Small Feature Replication in Melt Electrowritten Membranes to Model Kidney Proximal Tubule
Source: Adv Healthc Mater. 2024 Nov 7;14(1):2401800. doi: 10.1002/adhm.202401800 (PMC11694085; doi:10.1002/adhm.202401800)
Supplement: Supplementary file 1 — Supporting Information [file ADHM-14-0-s001.docx]

**Engineering Precision: Thermoforming for Small Feature Replication in Melt Electrowritten Membranes for Advanced Kidney Proximal Tubule Modeling**

*Marta G Valverde, Claudia Stampa Zamorano, Dora Kožinec, Laura Benito Zarza, Anne Metje van Genderen, Robine Janssen, Miguel Dias Castilho, Andrei Hrynevich, Tina Vermonden, Jos Malda, Mylene de Ruijter, Rosalinde Masereeuw, Silvia M Mihăilă**

** Corresponding author*

**Supporting information**

**Supplementary Table 1:** **Primary and secondary antibodies with dilutions**

**Supplementary Figure 1: Failed iterations of the thermoforming mold designs**

**Supplementary Figure 2: Characterization of MEW parameters**

**Supplementary Figure 3: MEW membranes before the thermoforming**

**Supplementary Figure 4: Proposed mechanism of the thermoforming**

**Supplementary Figure 5: Surface area gain enables larger area for cell culture**

**Supplementary Figure 6: Thermoforming with additional designs**

**Supplementary Figure 7: Growth of the ciPTECs on the thermoformed MEW membranes**

**Supplementary Figure 8: Polarization markers on the thermoformed MEW membranes**

**Supplementary Figure 9: MEW printing on alternative curved collectors**

**Supplementary Table 1:** **Primary and secondary antibodies with dilutions**

| Name | Dilution |
| --- | --- |
| **Primary antibodies** | |
| Goat monoclonal anti-collagen IV (1340-01 Southern Biotech) | 1:50 |
| Mouse monoclonal anti-α-tubulin (T6793, Sigma-Aldrich) | 1:150 |
| Rabbit monoclonal anti-Na^+^/K^+^-ATPase (gift from Prof. Jan Koenderink) | 1:100 |
| **Probes** | |
| AlexaFluor 647 phalloidin (A22283, Thermofisher Scientific) | 1:1000 |
| DAPI, dilactate 405 (D3571, Sigma-Aldrich) | 1:1000 |
| **Secondary antibodies** | |
| AlexaFluor 546 donkey anti-goat (A11056, Invitrogen) | 1:200 |
| AlexaFluor 488 goat anti-mouse (A11001, Invitrogen) | 1:200 |
| AlexaFluor 647 goat anti-rabbit (A21245, Invitrogen) | 1:200 |

**
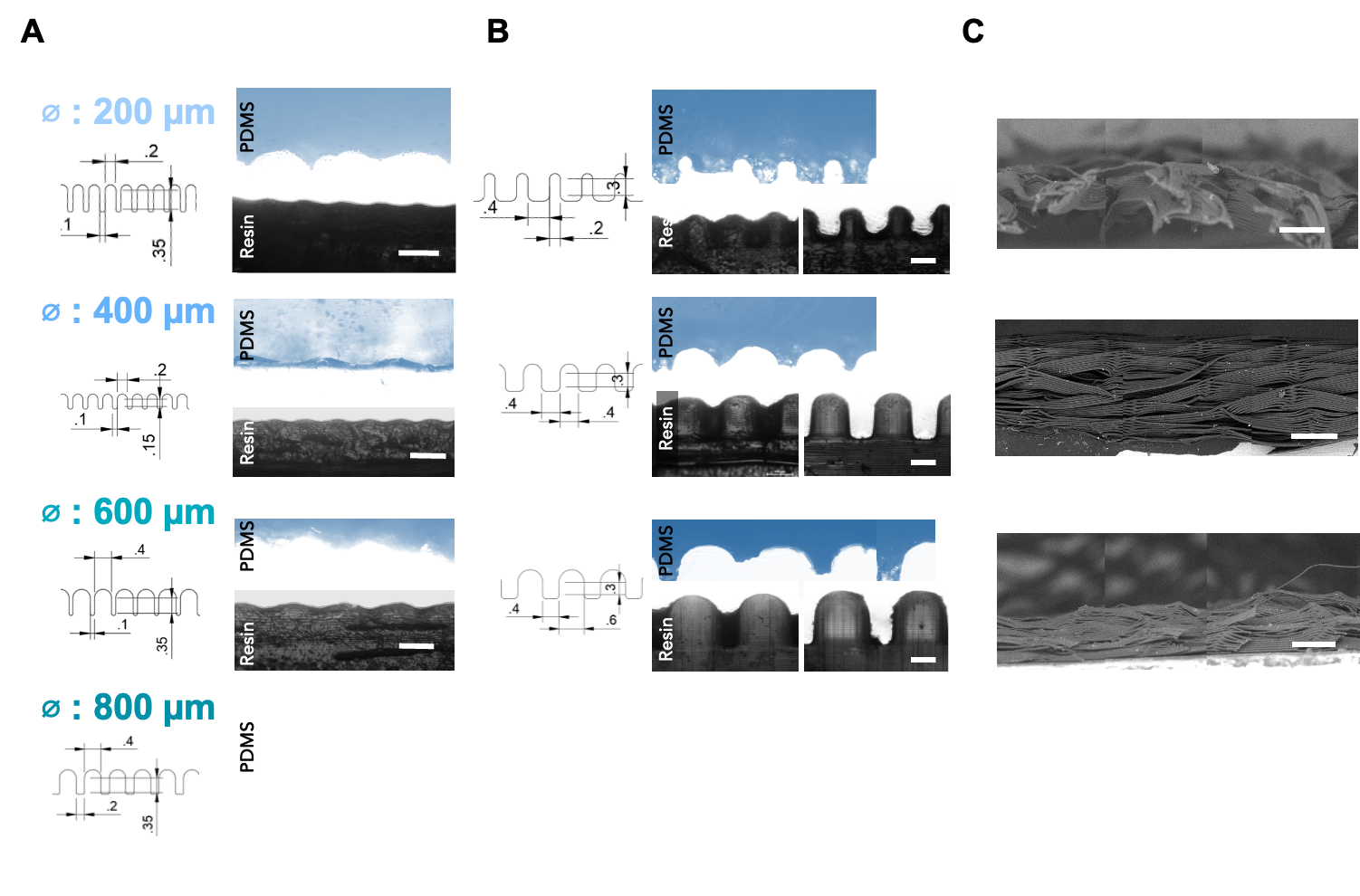
Supplementary Figure 1: Failed iterations of the thermoforming mold designs.** A) For 100 µm inter-groove spacing and 150 or 350 µm height the patters are not correctly printed and thus, the PDMS molds appear flat. B) Increasing the inter-groove spacing to 400 µm and 300 µm height improves printability of the resin slabs although there is fusion of the pillars for some regions. C) Composite of SEM images showing the membrane’s side view after thermoforming with incomplete molds. Scale bars 200 µm.

**
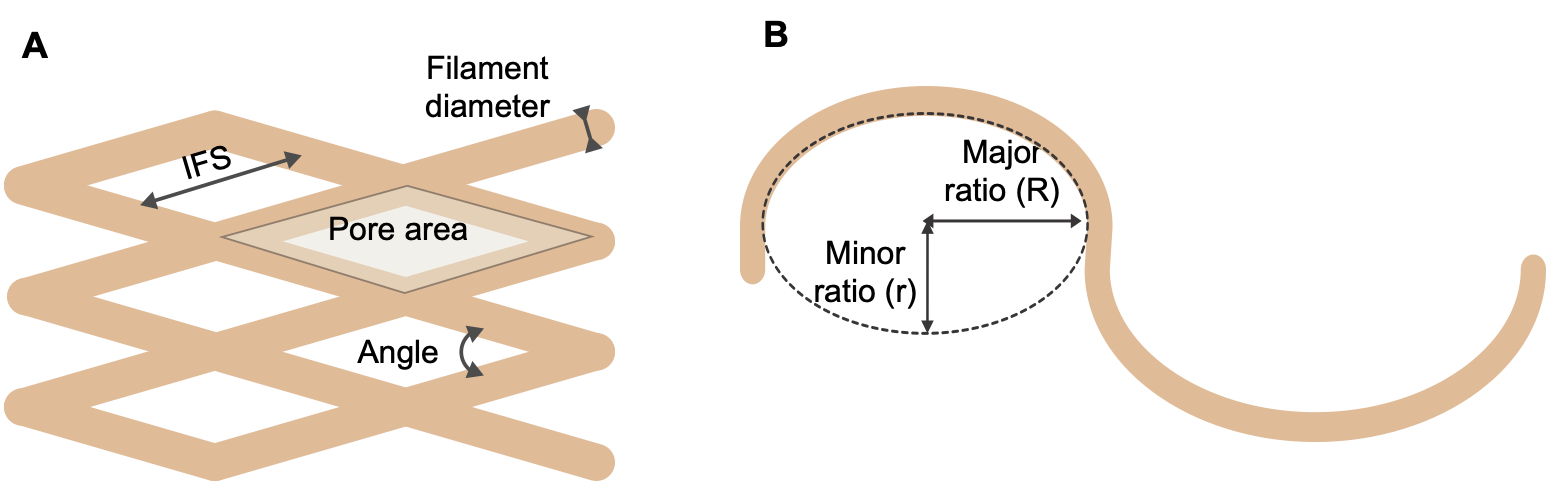
**

**Supplementary Figure 2: Characterization of MEW parameters.** A) Top view of the MEW, including interfiber spacing (IFS), interfiber angle, pore area, and filament diameter. (B) Side view of a thermoformed MEW membrane, including the mirror and major ratios. Schematics on CAD designs and pictures of the 3D printed resin molds (grey) and the PDMS casts (blue) for 200 µm, 400 µm, 600 µm and 800 µm in diameter

**
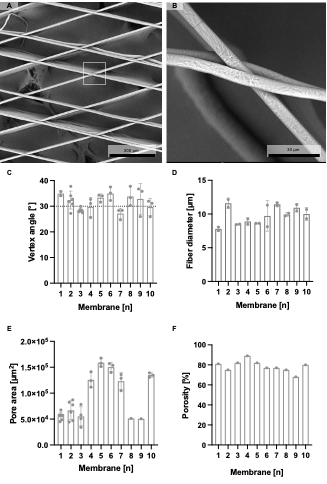
**

**Supplementary Figure 3: MEW membranes before the thermoforming.** A) SEM images of the diamond MEW membranes with a close-up view on the vertex (B). Characterization of the membranes’ parameters of interest before the thermoforming including winding angle (C), fiber diameter (D), pore area (E) and porosity (F). Plotted as Mean ± SD.

**
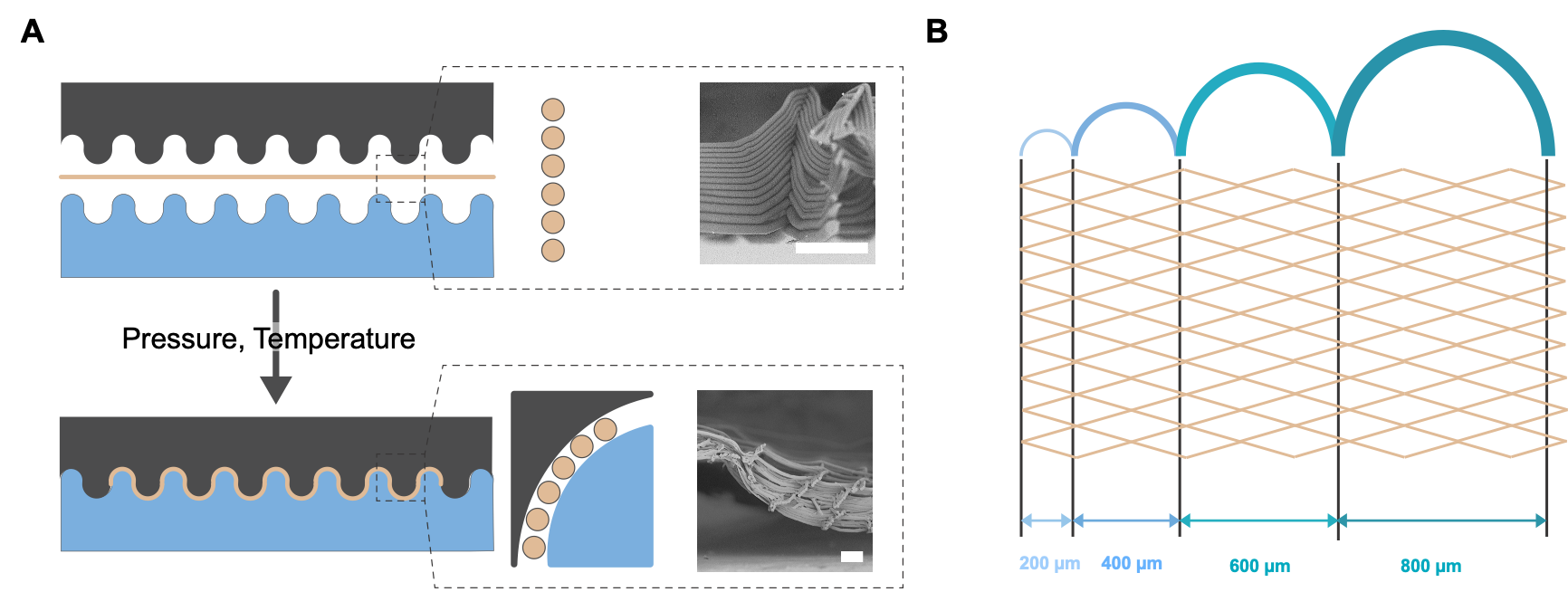
**

**Supplementary Figure 4: Proposed mechanism of the thermoforming.** (A) Schematic on the walls’ deformations during the thermoforming process. (B) Schematic on the number of pores per groove.

**
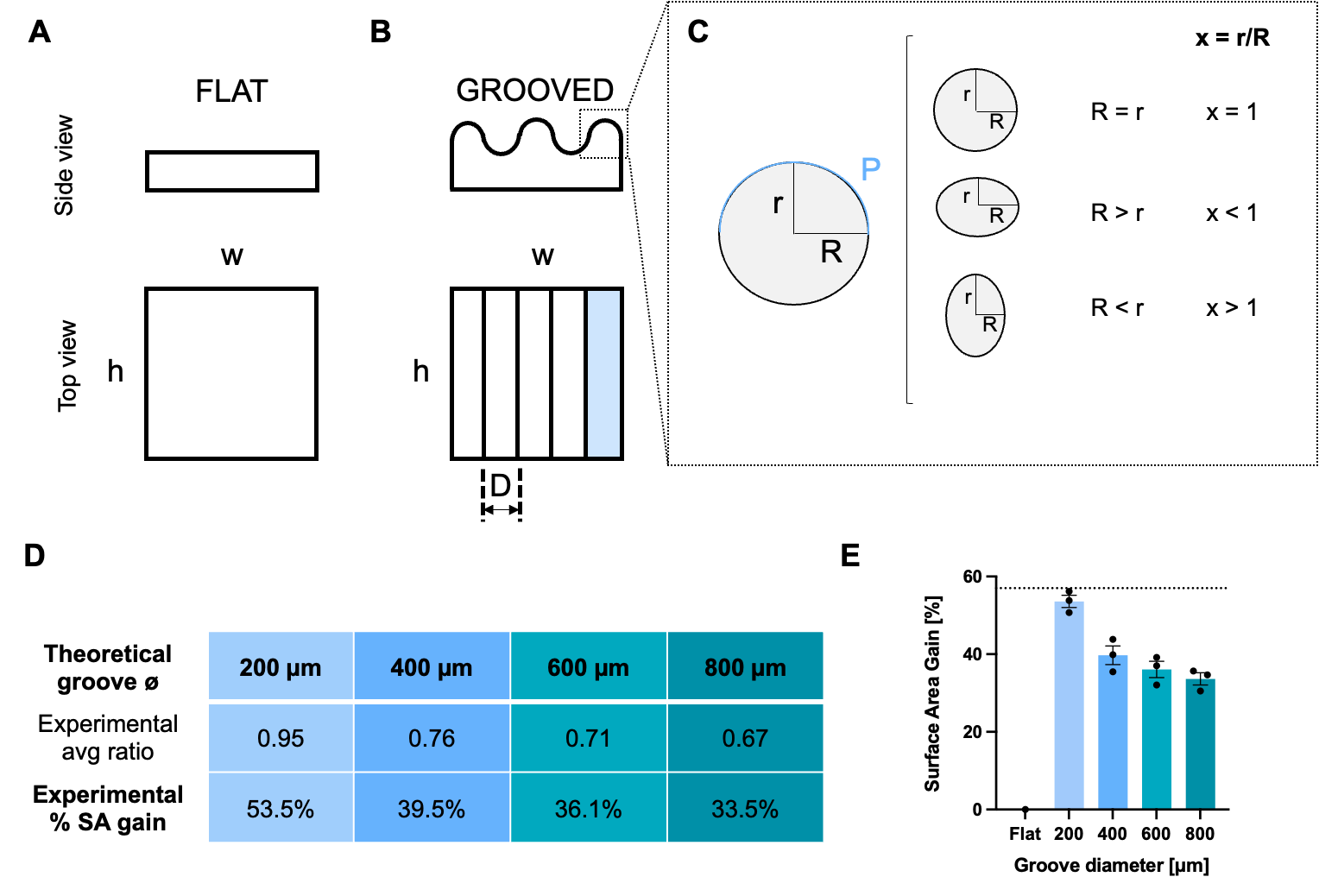
**

**Supplementary Figure 5: Surface area gain enables larger area for cell culture.** Schematics on the side and top views of the flat (A) and grooved (B) substrates with the same width (w) and height (h). (C) The grooves are ellipsoids with major and minor ratios (R, r, respectively), and a gained perimeter (P) for each undulation of the surface. The parameter x is defined as a ratio between the minor and the major ratio. The closer it is to one, the more circular the ellipsoid is. (D) Quantification of the real Surface Area (SA) gain for the thermoformed membranes resulting from combining equation 4 with the measured minor/major ratios, represented graphically in (E). Dotted line (Surface area gain = 57%) indicates the maximum theoretical value for a perfectly circular groove (minor/major ratio = 1).

**
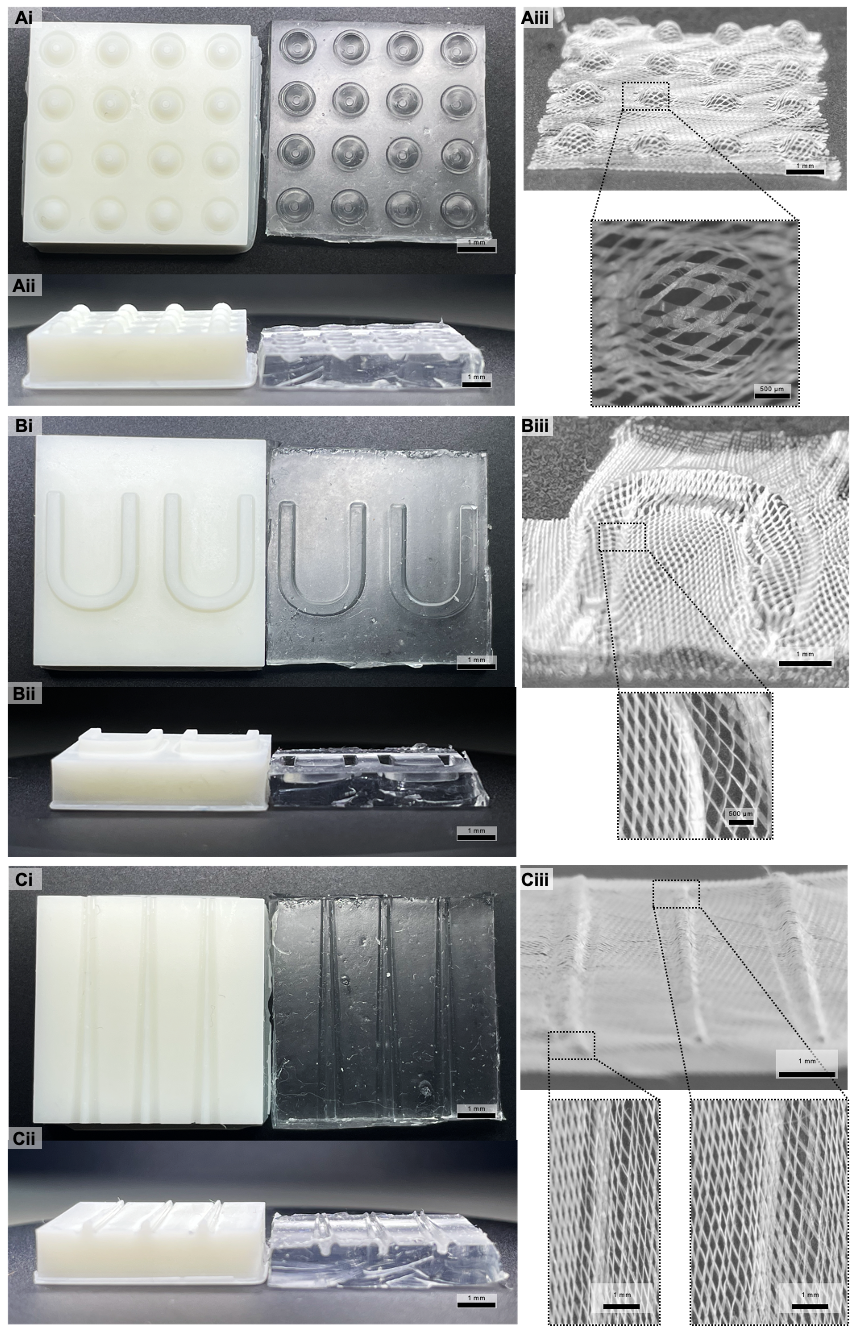
**

**Supplementary Figure 6: Thermoforming with additional designs.** (A) An array of half spheres (800 µm diameter), a horseshoe and three channels changing diameters (from 200 – 800 µm). Images correspond to: (i) top view of the 3D printed mold (white) and PDMS (transparent), (ii) side view of mold and PDMS, and (iii) angled view of the thermoforming membrane with zoomed in areas.


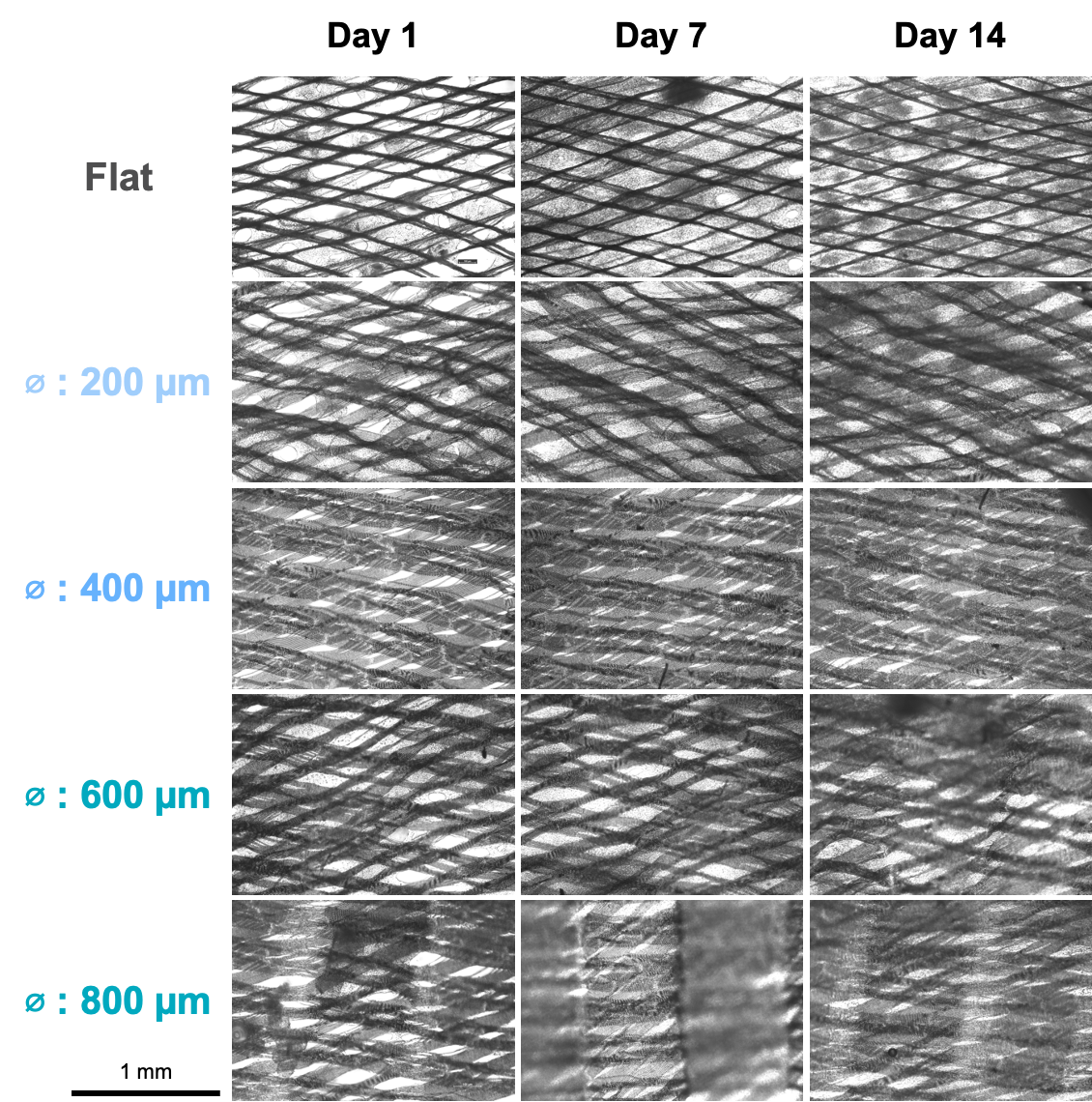


**Supplementary Figure 7: Growth of the ciPTECs on the thermoformed MEW membranes.** Bright field images of the membranes during culture 1 day after seeding, 7 days in culture and in day 14, before carrying out further experiments. The same membrane is imaged for the different time points. Blurred areas are out of focus grooves.

**
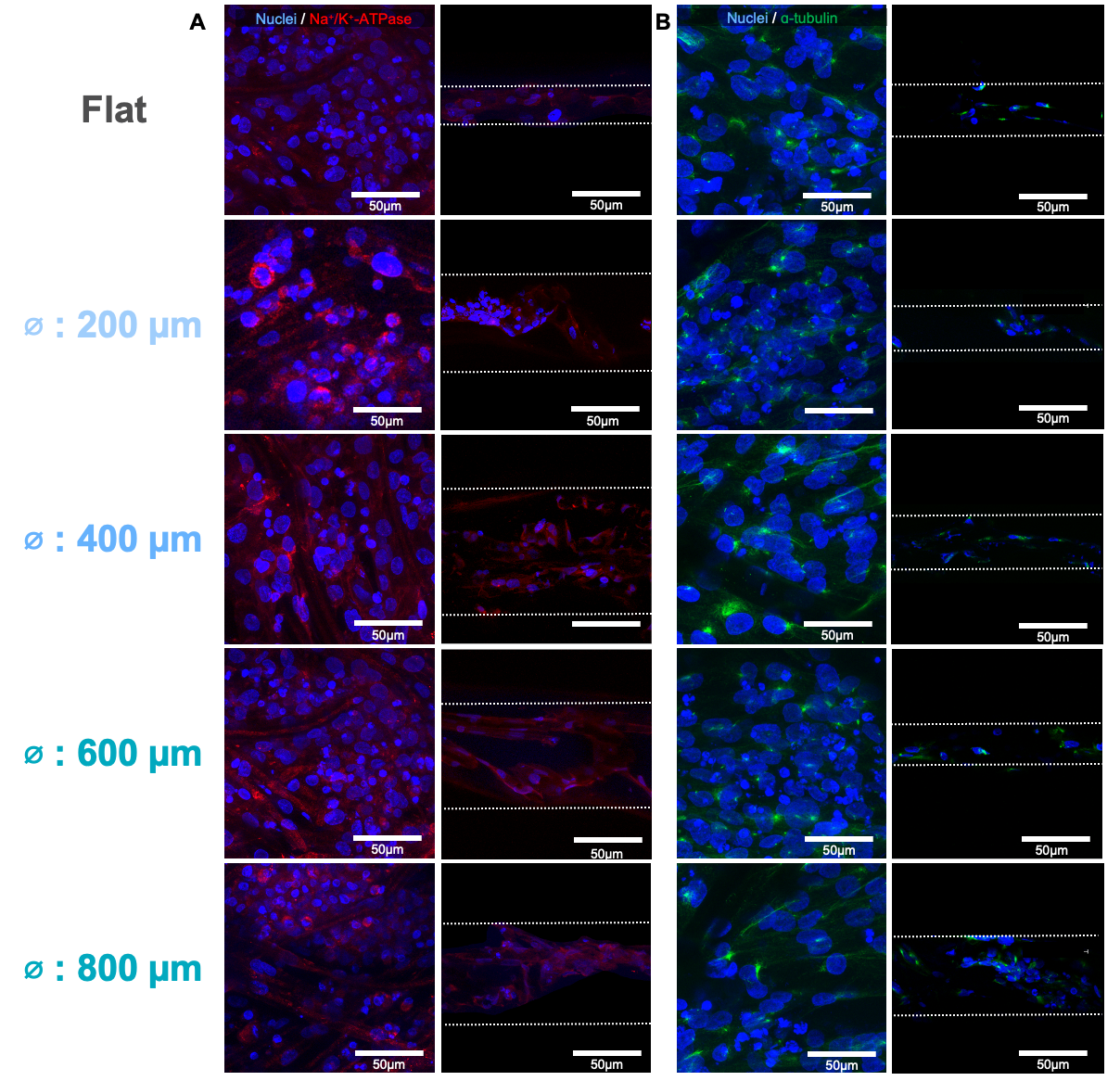
**

**Supplementary Figure 8: Polarization markers on the thermoformed MEW membranes.** Top and side views of nuclei / Na+/K+-ATPase (A) and nuclei / ɑ-tubulin (B). Adjacent images correspond to the same membranes, mounted flat and perpendicularly to the objective (63x).

**
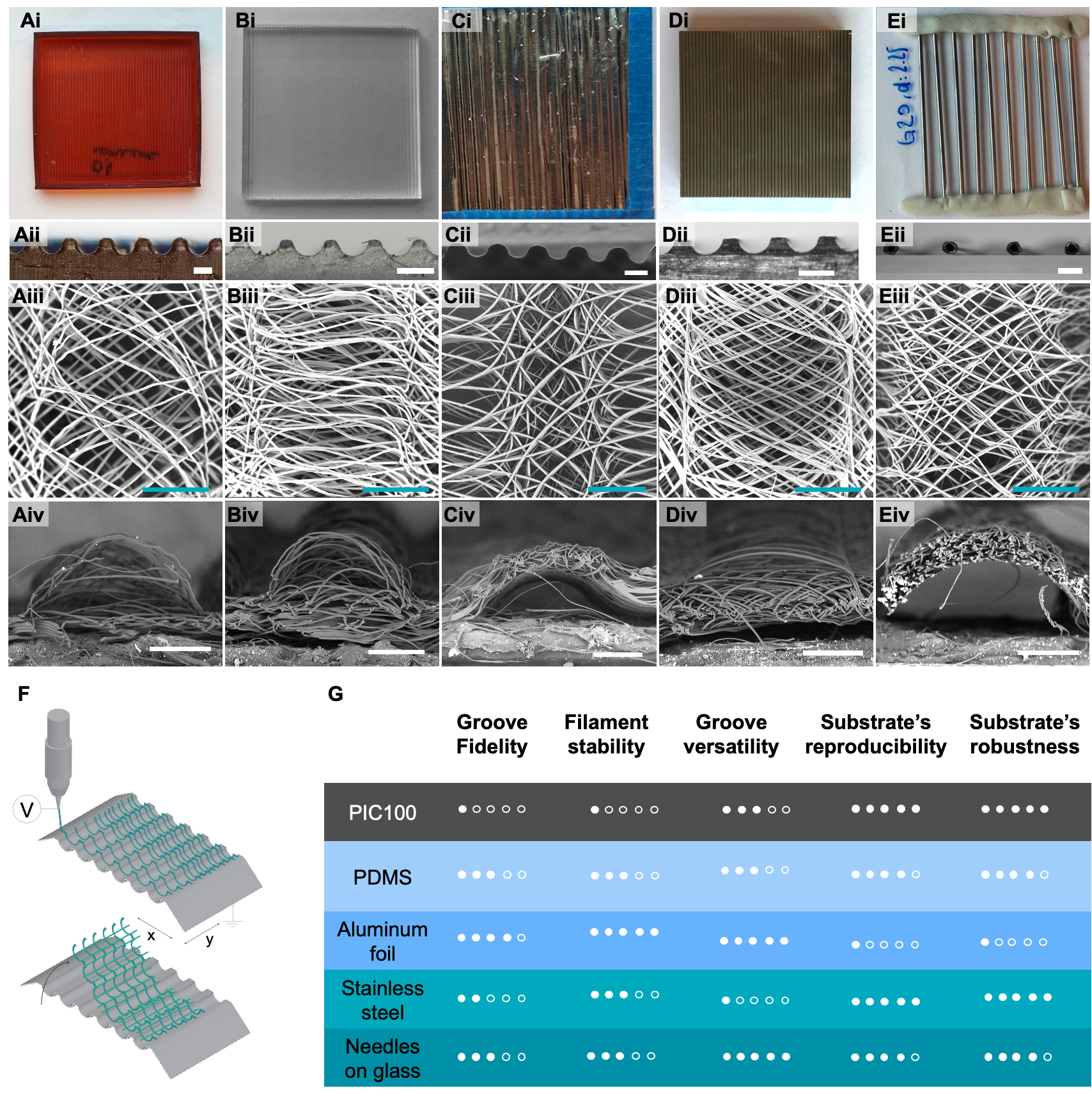
**

**Supplementary Figure 9: MEW printing on alternative curved collectors.** Prior data to the thermoforming study indicates that printing on top of a grooved collector does not ensure the faithful replication of the grooves nor the membrane’s design. The attempted collectors correspond to (A) 3D printed resin, (B) PDMS, (C) aluminum foil, (D) stainless steel, and (E) needles on glued to glass. For the upper panel (i) top view of the collector, (ii) side view of the collector, and SEM images of the membranes printed from the top (iii) and the side (iv). (F) Schematics on the printing process. (G) Table indicating the main characteristics of the collectors used. Scale bars (ii): 1 mm. Scale bars (iii and iv): 300 µm.
